# Dental Legislation

**Published:** 1875-12

**Authors:** W. C. Barrett


### DENTAL REGISTER.

VOL. XXIX.] DECEMBER, 1875. [NO. 12.

### DENTAL LEGISLATION.

BY W. C. BARRETT.

*Extracts from the Annual Address read before the New York State Dental Society, held at Albany, N. Y., June 30th, 1875.*

While the other professions have been of slow growth, their history being almost co-eval with man's creation, dental practice is the outgrowth of a later progress, the product of a higher civilization. The necessity for its existence has come from our more artificial life, and is a concomitant of an advanced culture. But though we may not claim that we were a profession since early antiquity, it does not necessarily follow that we are *parvenus;* for wherever man has reached a high state of enlightenment, there has been our home. Whenever the arts have flourished and men have become refined and therefore artificial, then have we had an existence. In the time of that early civilization when, six thousand years ago, that wonderful race of people existed in Egypt, with a knowledge of art and of many of the sciences far transcending that of their descendents to-day, among that people who have left monuments of their proficiency that in some respects put to shame the artistic achievements and study of the present time, there dentistry was known and respected with the other professions. In that later levantine civilization, when Christianity had not as yet thrown its light upon the world, but when tired of wars and slaughter and barbarism, men were cultivating the æsthetic tastes which raised so many noble monuments to mark the high tide of thought and artistic enlightenment, then was our profession not unknown ; and though the origin of modern dentistry is yet in full view, we can trace our remote lineage far back into antiquity. If however, despite these facts, we be reproached with our recent origin, we may reply that the youngest son is no less the brother because his years are fewer.

If we would take the place which of right belongs to us, we must come up to the high plane occupied by the other professions. When, possessed of equal learning, we aspire to equal recognition, we must first take equally firm ground, and give security that we will not recede from that position. In but very few states have we an organized existence. There is no central point around which may crystallize the elements of a profession. We are generally an irresponsible jumble of learned and ignorant, capable and incapable, competent men and quacks, the latter greatly in preponderance, and this undisciplined mob has pressed forward and demanded a place beside the professions whose immunities have for centuries been jealously guarded from interlopers and charlatans. Instead of our exemplifying the true professional principle, and endeavoring to disseminate scientific truths and knowledge, it has until quite lately been the practice for dentists to jealously guard their operating rooms and laboratories from the prying inspection of their neighbors, and to hoard up the poor scraps of knowledge they had acquired, lest their professional brother might benefit thereby. Or more probably, the possessor of some fancied or real improvement straightway secured a patent upon it, and began to vend out his knowledge. Dental patents have done more to prevent our attaining self-respect and the respect of others, than any one thing. He is scarce worthy to be called a member of a liberal profession, who only studies the field of science that he may make it tributary to his own selfish gratification;—who has no love for original investigation for its own sake, but only pursues it because it is capable of being turned into a source of personal profit;—who would use Promethean fire, stolen from heaven, to cook his steak by, and turn Pegasus into a cart horse. This is not the conduct of him who is worthy to be called a member of a learned profession; whose study is that he may gain knowledge for the purpose of imparting it to his professional brother, that thus his beloved profession may, *as a whole*, be raised, and the scientific education of its members, as a class, be thereby perfected.

To bring about this professional feeling, and to establish an *esprit de corps*, we must become thoroughly organized. I believe the best way to do this is through legally established state societies, having some recognized status."

I am not of those who imagine that a horde of ignorant, unprincipled men, may by mere legislative enactment, become entitled to the respect usually accorded to men of scientific attainments. But I *do* believe that, having subdued a certain portion of the field of science and adopted it as our heritage, having thoroughly explored and cultivated it, some legal recognition is necessary that our title may be made good. Men are governed by and pay great respect to law, and when that great profession places its seal of approval upon our claims, we have advanced a long way toward the goal of our ambition. When we are properly recognized by the law, we have a positive status; we are under its protecting ægis; we have a criterion by which the standing of practitioners may be definitely determined. The other professions, observing that our aims are laudable, and that we are endeavoring to separate the chaff from the wheat and thus establish a reputable practice, will accord us their respect and assistance. We shall be aiding ourselves by regulating our practice, and benefiting the people by delivering them from the risk of falling into the hands of unprincipled pretenders.

In the consideration of Dental Legislation, men are apt at once to jump to the conclusion that it means prohibition, and this it is which has arrayed against it all the quacks and many of the qualified practitioners. A greater mistake could not be made. If interdiction of any class ever be allowable, it comes only as a subsequent. The first aim of all dental legislation should be ORGANIZATION. We ought not to commence with enactments against any particular class, till they have had time to bring forth works meet for repentance. We should strive to convince the ignorant and incapable that we are laboring for the benefit of all, and thus secure their good will, and not render ourselves liable to the charge of proscription. To this end, while not throwing down one bar to their admission into our societies, we should smooth the way to the obtaining a proper professional education. To purge ourselves of all the gross elements in our midst, requires time and persevering effort. To perfect an organization that shall have the authority and possess the confidence to make its decisions respected, is not the work of a day. When, however, the other professions see such a responsible head of a body of learned men who are devoted to the study of science in their own special field, a center which gives some tangible object of recognition, such acknowledgment will not long be delayed; and therefore it is, that legally established societies are competent to do what voluntary organizations can not, being governed by a law which can compel unanimity of action.

In a few of the states of our common country, has legislation been procured. It has been of three kinds: First, stringent inhibitory laws, like those of Ohio, New Jersey and Georgia. Even in these states, there is but slight attempts to enforce the strict provisions of the statute, save in

Ohio, where they have encountered much opposition. Yet I believe that greater success in this direction has here been attained than any where else. I am indebted to J. M. Porter, D. D. S., of Toledo, in that state, for some statistics that are interesting. In the year 1873, he found that the whole number of dentists practicing in Ohio was 433. Of this number, 99 or about 23 per cent were graduates. Of the remainder 142, or about 43 per cent had passed the examining board, while 192, or about 44 per cent of the whole had done neither. Of all the dentists of Ohio, 56 per cent were legally qualified to practice, while the remainder, nearly half, were living in open violation of the law. Yet I doubt if there be another state in the Union, which can make so favorable a showing.

The second kind of legislation is incorporation of the state societies under the general laws, or by some special enactment. Simple incorporation gives no special recognition and conveys with it no privileges, except the unnecessary one of ability to hold property, and the rather dubious capability of sustaining an action at law. The state societies of Tennessee, Kentucky and Kansas, are chartered under such laws.

The third class of legislation is, in stringency, between these two extremes, while in perfection of organization it is superior to either, and is well represented by the New York State enactment, which gave birth to the Dental Society of the State of New York. The object of the orginators of this law, was to thoroughly organize the profession of the state, establish for it some definite limits, secure perfect legal recognition, and institute a criterion by which the good might be distinguished from the bad, thus throwing the whole moral weight of the community for the one and against the other. So well has it succeeded in its work, especially in the country districts, that I propose to here give a review of its provisions, setting it up as a kind of example of this class of legislation.

The New York State Dental Law divides the state for dental purposes into eight districts, following the boundaries of the judicial divisions as establishing lines familiarly known, and districting the state into convenient and proper provinces for the purpose sought. In each district the law establishes a dental society, and eight delegates from each district dental society, elected to serve for the term of four years, together with an indefinite number of permanent members, of whom five may be elected each year, to be selected from among the eminent practitioners of the state, and two representatives from each dental college in the state, makes up the state society, which the law provides shall meet at least once a year in the Capitol at Albany. Its expenses are provided for by the levying of dues upon each district society, by the dues of the permanent members, and by the fees to be collected upon conferring its diploma. District societies are subordinate to this state society, and must make to it an annual report of their status and proceedings. Each district society elects a board of censors, before whom must appear every student in dentistry who proposes to commence its practice. Should he successfully pass the examination of the board, he becomes entitled to the diploma of the society and to membership therein, and is permitted to open an office ; but without such diploma it is made unlawful for any one to commence the practice of dentistry *subsequent to the passage of the law.*

Such persons only, of those who entered the profession subsequent to July 1st, 1868, as comply with the law, are to be considered as qualified to practice. For such as fail to conform, no penalties are provided, other than being constructively branded by the law as quacks. From each one of the eight district societies, a censor is elected by the state society, and these form the board of state censors, whose duty it is to meet once each year, at the time of the annual meeting of the state society, and as much oftener as is demanded by the applications of students. They make a thorough investigation of the qualifications of each applicant, and such as pass their examination they recommend to the state society, to whom is given authority to confer upon them the diploma and degree of "Master of Dental Surgery,"

(M. D. S.) when the candidate shall have paid the necessary fees and signed the requisite bond to walk worthy of his vocation.

These are the principal provisions of a law which has wrought a large amount of good, especially in the rural districts. Its advantages are obvious. The plan of organizing district societies and making them subordinate to the central head, secures the most perfect organization possible. The members of the profession in contiguous localities are banded together, and from their intimate connection with other district societies and the commingling of delegates at the meetings of the parent organization, close professional intimacies are formed, and ties which bind together the whole profession of the state. A membership is secured which is not dependent upon the thousand contingencies attending a mere voluntary association, and full attendance is assured. The society being a delegated body is composed of good men, and when it speaks it can do so as having authority. Being also in part composed of permanent members, the continued co-operation of the best men of the profession is secured, and there is added an element of greater stability. There has never been any opposition to the law, for no prohibitory action or proscription was threatened, and thus was secured the concurrence of all classes. There was no difficulty in obtaining the passage of the enactment, for there was nothing in it to arouse opposition in the legislature, and to-day the law and society are so strongly entrenched in the good will of the people, that further legislation might, if thought desirable, be easily procured. There has been no attempt at bluster; all has been done quietly but thoroughly, and while no astonishing revolution has overturned the existing order of things, the good effects of the law have been felt everywhere, and its originators have every year seen greater and still greater reason to congratulate themselves upon the original moderateness of their demands. If in the future it be thought advisable to press more stringent measures, the society having gained a firm foothold and having practically enforced previous legislation, will find it comparatively easy to obtain enactments establishing proper penalties for the infringement of the present statute.

The establishing the degree of Master of Dental Surgery was thought to be demanded by the needs of the profession. It was never contemplated that it should in any manner conflict with, or be a substitute for that of Doctor of Dental Surgery. It is not an evidence of scholastic training, nor was it ever intended that it should be proof of having passed through the curriculum of the colleges. But there is a large class of practitioners, who from limited means or from other causes, have been obliged to obtain the most of their professional education at home. Some of them are close students, and are in reality better qualified for the practice of dentistry than many who have spent a full term at college. From business and family causes, they are unable now to give the time necessary for attendance upon a dental school. This diploma gives a ready means of determining the status of such, for the examination has heretofore been most strict. It has been so far however, chiefly sought by those who possess the D. D. S., as an evidence that they are in regular and reputable exercise of their profession, for it is only granted to such as are in actual, honorable practice. It was thought in establishing it, that perhaps it would be adopted by such other states as had proper legislation, and be respected by all as an evidence of compliance with the terms of the law. It does not in any way relieve the student from the necessity of attendance upon a dental college, as the degree of Doctor of Dental Surgery only, indicates the possession of the necesary qualifications for the practice of dentistry, while this is proof that he is putting to a reputable use his dental education, and is in successful practice. This degree, analagous to the Licentiate of Dental Surgery of Canada, though requiring much higher qualifications in the candidate for its possession, has been a great incentive to study on the part of the younger members of the profession, and indeed many of the older graduates have found it necessary to brush up considerably anticipatory to making their appearance before the Board of Censors. Instances have been, where the candidate has been repeatedly rejected, only to go through another year's careful study and another trial. The Censors are elected for four years, and as they have usually been re-elected when their fitness was demonstrated, they become thoroughly familiar with their duties.

The law has secured for us some valuable prerogatives. It expressly entitles us to "all the privileges and immunities heretofore accorded to the medical profession:" Under it, in the cities, dentists claim exemption from jury duty, and professional fees when summoned as professional witnesses. The annual reports of the society are made to the Legislature and are printed and published as official documents, the same as are the reports of the state medical societies, and the various charitable institutions under the patronage of the state. But the greatest advantage gained through legal enactment, is in the full recognition of the claim of dentistry to an honorable position by the side of medicine, law and theology. When the time comes, and the profession in all the states is fully organized, then will be settled the question concerning the proper status of dentistry. There should be something like uniform legislation in every state! Not identical enactment, but while conforming to one model and aiming at the same result, the details may be varied to suit the necessities of each case.

The profession in a majority of the states, is fully awake to the necessities of some such work. I hope that many of those now in practice, will see the time when every state in the Union shall possess laws fully recognizing dentistry, and compelling complete organization by counties or districts, each owing allegiance to a well sustained state society. If the state be not large enough to support more than two district societies, the parent organization will still be a delegated body. My hopes go farther than this: I would have delegates from each state society meet as the supreme authority in professional matters; a NATIONAL ASSOCIATION, worthy the name, legally constituted, and working under such general laws as should give it the authority to make its decisions respected. Such an organization, composed of delegates from each state society, would be eminently a legislative body. It would be composed of the best men of the profession, and would have such an order of nationality about it, as would give a distinct and suggestive meaning to the phrase "American Dentistry." Such a consummation is eminently worthy the best efforts of every one who has any real love for his profession, and who desires to see it take the place which by right belongs to it. The officers and members of the Dental Society of the State of New York, will gladly co-operate with any one who desires to earnestly work in this field, and to this end they invite correspondence with such as are desirous of seeing dentistry occupy its proper position before the law, more especially in those states which have as yet no legal enactments for the properly regulating of our noble profession.

